# Time-dependent effects of hypothermia on microglial activation and migration

**DOI:** 10.1186/1742-2094-9-164

**Published:** 2012-07-09

**Authors:** Jung-Wan Seo, Jong-Heon Kim, Jae-Hong Kim, Minchul Seo, Hyung Soo Han, Jaechan Park, Kyoungho Suk

**Affiliations:** 1 Department of Pharmacology, Brain Science & Engineering Institute, CMRI, Kyungpook National University School of Medicine, 101 Dong-In, Daegu, Joong-gu, 700-422, South Korea; 2 Department of Physiology, Brain Science & Engineering Institute, Kyungpook National University School of Medicine, Daegu, South Korea; 3 Department of Neurosurgery, Brain Science & Engineering Institute, Kyungpook National University School of Medicine, Daegu, South Korea

**Keywords:** Hypothermia, Microglia, Cell migration, Neuroinflammation, Neuroprotection

## Abstract

**Background:**

Therapeutic hypothermia is one of the neuroprotective strategies that improve neurological outcomes after brain damage in ischemic stroke and traumatic brain injury. Microglial cells become activated following brain injury and play an important role in neuroinflammation and subsequent brain damage. The aim of this study was to determine the time-dependent effects of hypothermia on microglial cell activation and migration, which are accompanied by neuroinflammation.

**Methods:**

Microglial cells in culture were subjected to mild (33 °C) or moderate (29 °C) hypothermic conditions before, during, or after lipopolysaccharide (LPS) or hypoxic stimulation, and the production of nitric oxide (NO), proinflammatory cytokines, reactive oxygen species, and neurotoxicity was evaluated. Effects of hypothermia on microglial migration were also determined in *in vitro* as well as *in vivo* settings.

**Results:**

Early-, co-, and delayed-hypothermic treatments inhibited microglial production of inflammatory mediators to varying degrees: early treatment was the most efficient, and delayed treatment showed time-dependent effects. Delayed hypothermia also suppressed the mRNA levels of proinflammatory cytokines and iNOS, and attenuated microglial neurotoxicity in microglia-neuron co-cultures. Furthermore, delayed hypothermia reduced microglial migration in the Boyden chamber assay and wound healing assay. In a stab injury model, delayed local hypothermia reduced migration of microglia toward the injury site in the rat brain.

**Conclusion:**

Taken together, our results indicate that delayed hypothermia is sufficient to attenuate microglial activation and migration, and provide the basis of determining the optimal time window for therapeutic hypothermia. Delayed hypothermia may be neuroprotective by inhibiting microglia-mediated neuroinflammation, indicating the therapeutic potential of post-injury hypothermia for patients with brain damages exhibiting some of the inflammatory components.

## Introduction

Brain injury can be classified as direct disruption of the brain tissue and as secondary damage. The primary tissue damage that occurs at the moment of impact results in immediate necrotic cell death, impaired regulation of cerebral blood flow, and disruption of the blood–brain barrier (BBB) [1]. Secondary brain damage is defined as delayed injury including brain inflammatory responses such as resident immune cell activation, peripheral blood cell infiltration, and edema formation [2,3]. After brain injury, necrotic cell debris can stimulate an initiation of inflammation, activation of phagocytic cells, and synthesis of inflammatory mediators, such as cytokines and chemokines [4-6]. The inflammatory mediators contribute to neuronal cell death, BBB disruption, and reactive gliosis involving activation of microglia and astrocytes [7,8]. Microglial cells, resident immune cells of the central nerve system (CNS), play a critical role in neuropathology following brain injury. In activated microglial cells responding to the injury, migration and phagocytosis are promoted, and inflammatory mediators including superoxide anion, nitric oxide (NO), and inflammatory cytokines, such as interleukin-1 beta (IL-1β) and tumor necrosis factor alpha (TNF-α), are released [9,10].

Induced hypothermia is a therapeutic intervention to treat brain injuries [11,12]. The protective mechanisms of mild or moderate hypothermia include the reductions in excitatory neurotransmitters, apoptotic cell death, dysfunctional mitochondria, and suppression of protein synthesis [13,14]. Previous reports have shown that hypothermia inhibits microglia and brain inflammation, which may be the underlying mechanism of the neuroprotective effects of mild hypothermia [15-19]. Our previous work has also demonstrated that hypothermia decreases NO and cytokine generation by microglia [20-22]. Although hypothermic protection has been considered a result of the preservation of metabolic stores and suppression of several key events as outlined above, alternative protection mechanisms have been suggested, such as changes in gene expression and the upregulation of anti-apoptotic factors or a family of cold shock proteins [23-25]. Nevertheless, the protective mechanism of therapeutic hypothermia remains yet to be fully understood. Several reports have suggested that neurological outcomes of therapeutic hypothermia may depend on the duration or initiation of the cooling time. Earlier initiation of the cooling seems to give a better outcome [26-28]. However, optimal brain cooling conditions have yet to be defined.

In this study, we attempted to determine the optimal cooling conditions of therapeutic hypothermia using microglial cell culture as well as *in vivo* models by examining the time-dependent effect of hypothermia on: 1) microglial NO production and cytokine expression; 2) microglia-mediated neurotoxicity in a microglia-neuron co-culture model; and 3) migration of microglial cells in cultures as well as *in vivo*. Our results show that delayed hypothermia is sufficient to attenuate microglial activation, migration, and possibly subsequent neuroinflammation. Our results also provide the basis of the optimal time window for hypothermic treatment.

## Methods

### Reagents and cell culture

The following chemicals were obtained from Sigma Chemical Co. (St Louis, MO, USA): bacterial lipopolysaccharide (LPS) from *Escherichia coli* 0111:B4, hydrogen peroxide (H_2_O_2_), and sodium nitroprusside (SNP). The immortalized BV-2 murine microglial cells [29] were maintained in DMEM (Lonza, Walkersville, MD, USA) containing 5 % heat-inactivated fetal bovine serum (FBS) (Lonza) and gentamicin (50 μg/ml) (Gibco-BRL, Rockville, MD, USA) at 37 °C. Mouse primary microglia cultures were prepared by mild trypsinization from mixed glial cultures as previously described with minor modifications [30]. In brief, the forebrains of newborn ICR mice were chopped and dissociated by mechanical disruption using a nylon mesh. After *in vitro* culturing for 10 to 14 days in DMEM supplemented with 10 % FBS, 100 U/ml penicillin, and 100 μg/ml streptomycin (Gibco-BRL), mixed glial cultures were incubated with a trypsin solution (0.25 % trypsin, 1 mM ethylenediamine tetraacetic acid (EDTA) in Hank's balanced salt solutions) diluted 1:4 in PBS containing 1 mM CaCl_2_ for 30 to 60 minutes. This resulted in the detachment of an upper layer of astrocytes in one piece, whereas microglia remained attached to the bottom of the culture flask. The detached layer of astrocytes was aspirated, and the remaining microglia were used for experiments. Primary astrocyte cultures were prepared from mixed glial cultures by differential shaking as previously described [31]. The B35 rat neuroblastoma cell line (ATCC, CRL-2754) [32] stably expressing enhanced GFP (EGFP) was maintained in DMEM containing 10 % heat-inactivated FBS, penicillin (100 U/ml) and streptomycin (100 μg/ml) at 37 °C under a humidified atmosphere with 5 % CO_2_. Primary cultures of dissociated cerebral cortical neurons were prepared from embryonic day 20 (E20) ICR mice as described previously [33]. Cells were exposed to hypothermic conditions by placing them into cell culture incubators set to 33 °C or 29 °C. The actual hypothermic condition was confirmed by measuring the temperature of the media.

### Hypoxia

Hypoxia was implemented as previously described with minor modifications [34]. To induce hypoxic conditions, cells were washed three times with deoxygenated PBS and DMEM containing FBS, and incubated in an anaerobic chamber (atmosphere of 95 % N_2_/5 % CO_2,_; O_2_ tension < 5 %) (Anaerobic System 1025, Forma Scientific Inc., Marietta, OH, USA) for the indicated time period. Reoxygenation was performed by exchanging the media with a fresh DMEM, and cells were transferred into a regular normoxic incubator (95 % air, 5 % CO_2_) and the incubation continued for the indicated time periods.

### Nitrite quantification

NO_2_ concentrations in culture supernatants were measured to assess NO production in microglial cells using the Griess reagent as previously described [35]. Fifty microliters of sample aliquots were mixed with 50 μl of Griess reagent (1 % sulfanilamide/0.1 % naphthylethylene diamine dihydrochloride/2 % phosphoric acid) in a 96-well plate. The absorbance at 550 nm was measured on a microplate reader. NaNO_2_ was used as the standard to calculate the NO_2_ concentrations.

### Cell viability test

Cell viability was determined by the 3-(4, 5-dimethylthiazol-2-yl)-2, 5-diphenyltetrazolium bromide (MTT) assay as previously described [36]. BV-2 microglial cells, primary microglia cultures, or B35-EGFP cells were seeded in triplicate at a density of 3 to 5 x 10^4^ cells/well on a 96-well plate, and then treated as indicated. MTT was added to each well, and the cells were incubated for four hours at 37 °C. After discarding the culture media, dimethyl sulfoxide (DMSO) was added to dissolve the formazan dye. Absorbance at 570 nm was measured with a microplate reader (Anthos Labtec Instruments, Wals, Austria).

### Microglia/neuron co-cultures

Microglia/neuroblastoma co-culture was performed using BV-2 microglial cells or primary microglial cells (1.5 x 10^4^) and B35 neuroblastoma cells (3.75 x 10^4^) stably expressing EGFP (B35-EGFP) in a 96-well plate. LPS (100 ng/ml)-stimulated BV-2 or primary microglial cells were co-cultured with B35 neuroblastoma cells under normothermic or hypothermic conditions. After 24 hours of incubation, viable B35-EGFP cells were counted to measure microglial neurotoxicity. Primary microglia and primary neuron co-cultures were done using culture inserts as described previously [37]. Cortical neuron cells were plated at a density of 5 x 10^4^ cells per well in 150 μl of medium in 96-well companion plates (Nunc, Roskilde, Denmark), and allowed to settle at 37 °C in 95 % air/5 % CO_2_. In a separate plate, primary microglia cells were plated at a density of 3 x 10^4^ cells per insert in 80 μl of medium in cell culture inserts (0.2 μm pore size) and allowed to settle at 37 °C overnight in 95 % air/5 % CO_2_. Cell culture inserts containing the primary microglia cells were then inserted into the wells containing the cortical neuron cells before LPS and hypothermic treatment. For the collection of microglia conditioned media, primary microglia cells were plated at a density of 5 x 10^4^ cells per well in 150 μl of medium in 96-well plates, and stimulated with LPS under either hypothermic or normothermic condition. Conditioned medium was collected from the cells as previously described [38], and centrifuged at 1,200 rpm for two minutes to remove cellular debris. Primary microglia conditioned medium was added to cortical neuron cells that were plated at a density of 5 x 10^4^ cells per well in 96-well plates. Cortical neuron cells were further incubated for 24 hours in 95 % air 5 % CO_2_ under either hypothermic or normothermic condition before viability measurement.

### Traditional or real-time reverse transcription-PCR

Total RNA was extracted from cells or tissues with the TRIzol reagent (Invitrogen, Carlsbad, CA, USA), according to the manufacturer’s instructions. Reverse transcription was done with Superscript II (Invitrogen) and oligo(dT) primers. PCR amplification, using specific primer sets, was carried out at an annealing temperature of 55° to 60 °C for 20 to 30 cycles. The PCR was performed with a DNA Engine Tetrad Peltier Thermal Cycler (MJ Research, Waltham, MA, USA). For the analysis of the PCR products, 10 μl of each PCR product were electrophoresed on a 1 % agarose gel and detected under UV light. GAPDH was used as an internal control. The real-time PCR was done with a One Step SYBR® PrimeScript™ RT-PCR Kit (Perfect Real Time; Takara Bio Inc., Tokyo, Japan) according to the manufacturer’s instructions, followed by detection with an ABI Prism® 7500 Sequence Detection System (Applied Biosystems, Foster City, CA, USA). Nucleotide sequences of the primers were based on published cDNA sequences (Table 1).

**Table 1:** DNA sequences of the primers used for RT-PCR.

**cDNAs**	**RT-PCR methods**	**Primer sequences**	**GenBank accession No.**	**RT-PCR product size (bp)**
Mouse	Traditional	**Forward, 5’-GCA ACT GTT CCT GAA CTC-3’**	**NM_008361**	**382**
*il-1β*		**Reverse, 5’-CTC GGA GCC TGT AGT GCA-3’**		
Mouse	Real-Time	**Forward, 5’-AGT TGC CTT CTT GGG ACT GA-3’**	**NM_008361**	**102**
*il-1β*		**Reverse, 5’-TCC ACG ATT TCC CAG AGA AC-3’**		
Mouse	Traditional	**Forward, 5’-CCC TTC CGA AGT TTC TGG CAG CAG C-3’**	**NM_010927**	**497**
*inos*		**Reverse, 5’-GGC TGT CAG AGC CTC GTG GCT TTG G-3’**		
Mouse	Real-Time	**Forward, 5’-GCC ACC AAC AAT GGC AAC A-3’**	**NM_010927**	**103**
*inos*		**Reverse, 5’-CGT ACC GGA TGA GCT GTG AAT T-3’**		
Mouse	Traditional	**Forward, 5’-CAT CTT CTC AAA ATT CGA GTG ACA A-3’**	**NM_013693**	**411**
*tnf-α*		**Reverse, 5’-ACT TGG GCA GAT TGA CCT CAG-3’**		
Mouse	Real-Time	**Forward, 5’-ATG GCC TCC CTC TCA GTT C-3’**	**NM_013693**	**104**
*tnf-α*		**Reverse, 5’-TTG GTG GTT TGC TAC GAC GTG-3’**		
Mouse	Real-Time	**Forward, 5’- GCC CTC TCT CTC CTC TTG CT -3’**	**NM_013652**	**196**
*ccl4*		**Reverse, 5’- GTC TGC CTC TTT TGG TCA GG -3’**		
Mouse	Real-Time	**Forward, 5’- CGA CTG TTG CCT CTC GTA CA -3’**	**NM_016960**	**177**
*ccl20*		**Reverse, 5’- AGG AGG TTC ACA GCC CTT TT -3’**		
Mouse	Real-Time	**Forward, 5’-AAG TGC TGC CGT CAT TTT CT-3’**	**NM_021274**	**186**
*cxcl10*		**Reverse, 5’-GTG GCA ATG ATC TCA ACA CG-3’**		
Mouse	Real-Time	**Forward, 5’-AGT TGC CTT CTT GGG ACT GA-3’**	**NM_031168**	**159**
*il-6*		**Reverse, 5’-TCC ACG ATT TCC CAG AGA AC-3’**		
Mouse	Real-Time	**Forward, 5’- AAG GAC CAG CTG GAC AAC AT -3’**	**NM_010548**	**230**
*il-10*		**Reverse, 5’- TTT TCA CAG GGG AGA AAT CG 3’**		
Mouse	Real-Time	**Forward, 5’- CAT CGA TGA GCT GAT GCA GT -3’**	**M86672**	**163**
*il-12*		**Reverse, 5’- CAG ATA GCC CAT CAC CCT GT -3’**		
Rat/mouse	Traditional	**Forward, 5’-ACC ACA GTC CAT GCC ATC AC-3’**	**NM_008084**	**452**
*gapdh*		**Reverse, 5’-TCC ACC ACC CTG TTG CTG TA-3’**		
Mouse	Real-Time	**Forward, 5’- TGG GCT ACA CTG AGC ACC AG -3’**	**NM_008084**	**171**
*gapdh*		**Reverse, 5’- GGG TGT CGC TGT TGA AGT CA-3’**		
Rat	Traditional	**Forward, 5’-CTT TCA TCA CAC AGG ACA GG-3’**	**NM_031512.2**	**228**
*il-1β*		**Reverse, 5’-GTG ATG TTC CCA TTA GAC AGC-3’**		
Rat	Traditional	**Forward, 5’-GAC GCC CCG GCC TTC CAA ATA AAT-3’**	**NM_012675.3**	**800**
*tnf-α*		**Reverse, 5’-GCT GCC CCG ACT ATG TGC TCC TCA-3’**		
Rat	Traditional	**Forward, 5’-CAG ACA GTT TCT GGT CGA TGT CAT GA -3’**	**D44591**	**230**
*iNOS*		**Reverse, 5’-CTG CAT GGA ACA GTA TAA GGC AAA C -3’**		

bp, base pair; RT-PCR, reverse transcriptase-polymerase chain reaction.

### Measurement of the production of reactive oxygen species (ROS)

Production of microglial H_2_O_2_ was measured by the Amplex Red-horseradish peroxidase method (Molecular Probes, Eugene, OR, USA). Horseradish peroxidase (HRP, 0.1 unit/ml) catalyzes the H_2_O_2_-dependent oxidation of non-fluorescent Amplex Red (50 μM) to fluorescent resorufin red [39]. Microglial cells were plated in 24-well culture plates at a density of 2 x 10^5^ cells/well, and treated with LPS (100 ng/ml) for six hours under normothermic or hypothermic conditions. Afterwards, cells were detached and resuspended in N-2-hydroxyethylpiperazine-N'-2-ethanesulfonic acid (HEPES buffer, Gibco-BRL) (2 x 10^4^ microglial cells in 50 μl), to which 50 μl of a mixture containing Amplex Red reagent and HRP were added in the wells of a 96-well fluorescence plate. After one hour of incubation at 37 °C, fluorescence measurements were carried out at excitation/emission wavelengths of 544/590 nm. H_2_O_2_ of known concentrations was used to construct standard curves. ROS visualization was performed as described previously [40]. Microglial cells plated in 24-well culture plates were treated under normothermia or hypothermia as described above, and incubated for 30 minutes at 37 °C in media containing 10 μM of 5-(and 6-)chloromethyl-2’,7’-dichlorodihydrofluorescein diacetate (CMH_2_DCFDA; Molecular Probes). The cultures were washed twice with PBS and subjected to fluorescence microscopic analysis. DCFDA-positive cells were counted from three randomly chosen microscopic fields (Olympus BX50). These three measurements were averaged and regarded as a data point.

### *In vitro* cell migration assay

Cell migration was determined with a 48-well Boyden chamber (NeuroProbe, Gaithersburg, MD, USA) according to the manufacturer’s instructions [41]. Cells (1 x 10^4^ cells per well) were added to the upper chamber separated from the bottom wells by polyvinylpyrrolidone-free polycarbonate filters (8 μm pore size; 25 x 80 mm; NeuroProbe). Cells were incubated under normothermic or hypothermic conditions for the indicated time period. At the end of the incubation, non-migrating cells on the upper side of the membrane were removed with a cotton swab. Migrated cells on the lower side of the membrane were fixed with methanol for ten minutes and stained with Mayer’s Hematoxylin (Dakocytomation, Glostrup, Denmark) for 20 minutes. Photomicrographs of five randomly chosen fields were taken (Olympus CK2; Tokyo, Japan) and cells were enumerated to calculate the average number of cells that had migrated. All migrated cells were counted, and the results are presented as the mean ± SD of triplicates. The scratch wound healing assay was performed as previously described [42]. In brief, a scratch wound was created by using a 200 μl pipette tip on confluent cell monolayers in 24-well culture plates, and placed into DMEM containing 5 % FBS and 50 μg/ml gentamicin. Cells were incubated under normothermic or hypothermic conditions during migration of the monolayer into the cleared wound area. The wound area was observed by microscopy (Olympus BX50). The relative cell migration distance was determined by measuring the wound width and subtracting this from the initial value as previously described: cell migration distance = initial wound width - wound width at the time of measurement [43]. Three non-overlapping fields were selected and examined in each well (three wells per experimental group). The results are presented as an average fold increase of the migration distance.

### *In vivo* cell migration assay

#### Animal surgery

Male Sprague–Dawley rats, with weights ranging from 300 to 350 g were purchased from the Samtako Co. (Osan, Korea). Rats were housed in individual cages at 22 °C on a 12 hour day/night cycle and freely allowed to access food and water. All surgical procedures were carried out according to the guidelines of the Kyungpook National University Animal Experiment Ethics Committee. Anesthetization was performed by intraperitoneal injection of tiletamine/zolazepam (30 mg/kg (Zoletil; Virbac Laboratories, Carros, France)) and xylazine (10 mg/kg (Rompun; Bayer, Puteaux, France)), and animals were positioned in a stereotaxic apparatus (Stoelting, Wood Dale, IL, USA). Rats were placed on a homeothermic heat blanket (Harvard Apparatus Co., South Natick, MA, USA) at 37 °C to maintain normal body temperature during surgery. The skull was exposed by a skin incision, and small burr holes were drilled through the skull. The guide cannula was implanted at the stereotaxic coordinates of −2 mm anterior to the bregma, +3 mm lateral to the bregma, and −1 mm below the dura using a 22 G needle and cemented.

#### Cooling coil

Local cooling was performed using a cooling coil as previously described with slight modification [44]. Briefly, hypodermic tubing (26 G) was bent into a spiral pattern measuring 4 mm in diameter and 0.5 mm in thickness. The coil was connected to the tube combined with a 0.46-mm internal diameter silastic tubing (Dow Corning Corp., Midland, MI, USA) and a 23 G scalp vein set. Cold water (4 °C) was perfused from the homoeothermic device, and the water circulation was controlled by a peristaltic pump. In order to allow animals to move freely, the water was infused through a stainless swivel (Instech Laboratories, Inc., Plymouth Meeting, PA, USA). The cooling device was surgically placed into a space between the temporalis muscle and the left side of the skull. Local cooling was estimated by measuring brain temperature after implanting the sterilized metal probe through a guide cannula. Ipsilateral brain temperature was locally maintained at 33 °C for 66 to 78 hours (data not shown), while the temperature in the contralateral side was maintained at normal body temperature. The device and guide cannula were well cemented carefully.

#### Focal stab injury and hypothermic strategies

To investigate the effect of hypothermia on microglial migration *in vivo*, focal stab injury was given by locating a 26 G needle in the cortical area of the brain through 22 G guide cannula at −2 mm below the dura mater. Hypothermia (33 °C) using cold water circulation was initiated either at six hours before or after focal stab injury and maintained for 72 hours. The needles were kept in place for five minutes and then removed. The guide cannula was covered with paraffin, and the animals were returned to their cages before sacrifice after 72 hours.

#### Microglia immunohistochemistry

Rats were anesthetized with diethyl ether and perfused with 4 % paraformaldehyde (PFA) diluted in 0.1 M PBS after transcardial perfusion with cold saline. Brains were post-fixed by using 4 % PFA for three days, and then cryo-protected with 30 % sucrose solution for three days. Three animals were used for each experimental group. The fixed brains were embedded in optimal cutting temperature (OCT) compound (Tissue-Tek, Sakura Finetek, Japan) for frozen section and then cut into 12-μm-thick horizontal sections. For immunohistochemistry, the sections were washed briefly and blocked with 1 % BSA and 5 % normal donkey serum. After washing with PBS, the sections were incubated at 4 °C overnight with the rabbit polyclonal anti-Iba-1 antibody (1:500 dilution; WAKO, Japan). The sections were then incubated with donkey FITC- or Cy3-conjugated anti-rabbit IgG antibody (1:200 dilution; Jackson Immunoresearch Laboratories; West Grove, PA, USA). The sections were mounted on 4',6-diamidino-2-phenylindole (DAPI)-containing gelatin solution. Data acquisition and immunohistological intensity measurement of Iba-1 staining was performed with a NIH image J program (NIH Image; Bethesda, MD, USA) as described previously [33]. In brief, tiled images of each section were captured with a CCD color video camera (Olympus D70). A composite of the images (4000 x 3000 pixels each; 1 pixel = 2.5 μm x 2.5 μm) was then constructed for each section with Photoshop CS3 version. The images were binary thresholded at 50 % of the background level, and the particles were then converted to a sub-threshold image area with a size less than 300 and larger than 5 pixels, which was judged as Iba-1 positive cells. To count the Iba-1 positive cells, three concentric circles were placed in the peri-region of the injury site in the sub-threshold image of the six independent sections. The cells in the three circles were counted and statistically analyzed.

#### 5-Bromo-2'-deoxyuridine assay

5-Bromo-2'-deoxyuridine (BrdU, 200 mg/kg, Sigma) was administered intraperitoneally. The rats were sacrificed at three days after the BrdU injection. The brain tissues were horizontally sliced and prepared for immunohistochemistry as described above. Then, DNA denaturation was performed by incubating with 1 M HCl for 30 minutes at 25 °C. The tissues were sequentially incubated with rat monoclonal anti-BrdU antibody (1:200, Serotec, Kidlington, UK) and FITC-conjugated anti-rat IgG antibody (1:200).

### Statistical analysis

Statistical comparisons between different treatments were done by either a Student’s *t*- test or one-way ANOVA with Dunnett’s multiple-comparison tests with the SPSS program (version 14.0 K) (SPSS Inc., Chicago, IL). Differences with a value of *P* < 0.05 were considered to be statistically significant.

## Results

### Inhibitory effect of hypothermia on microglial NO production

**Figure 1:**
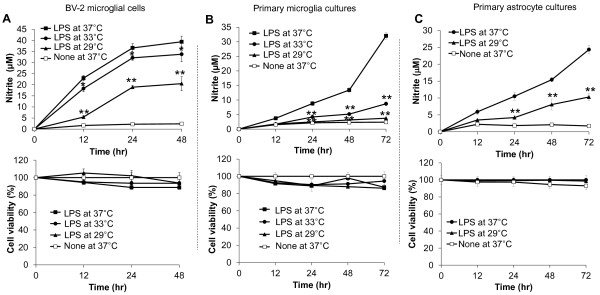
**Inhibition of microglial and astrocytic NO production by hypothermia.** BV-2 microglial cells (**A**), primary microglia cultures (**B**), primary astrocyte cultures (**C**) (5 x 10^4^ cells per well in a 96-well plate) were incubated with 100 ng/ml of LPS under hypothermic (33 °C, 29 °C) or normothermic (37 °C) conditions for 0 to 72 hours. The amounts of nitrite in the culture media were measured with the Griess reagent (upper). Cell viability was examined by MTT assays and the results were expressed as the percentage of surviving cells over the controls (lower). Results are the mean ± SD (n = 3). *P < 0.05, **P < 0.01; significantly different from the LPS-treated group at the same time point under normothermic condition (37 °C). LPS, lipopolysaccharide; MTT, 3-(4, 5-dimethylthiazol-2-yl)-2, 5-diphenyltetrazolium bromide; n, number.

To examine the effect of hypothermic conditions on NO synthesis in activated microglia cells, BV-2 mouse microglial cells were treated with TLR4 ligand LPS (100 ng/ml) under normothermic (37 °C) or hypothermic conditions (33 °C, 29 °C) for 0 to 48 hours, and nitrite production was measured. Hypothermic conditions led to a decrease in NO production in LPS-activated BV-2 microglial cells compared to normothermia (Figure 1A). The effect of 29 °C was greater than that of 33 °C. The inhibitory effect of hypothermia was similarly observed in primary microglial cells (Figure 1B) and primary astrocyte cultures (Figure 1C). The hypothermic condition did not significantly affect cell viability of either BV-2 microglial cells, primary microglial cells, or primary astrocyte cultures (Figure 1).

### Time-dependent effects of hypothermia on microglial NO production

**Figure 2:**
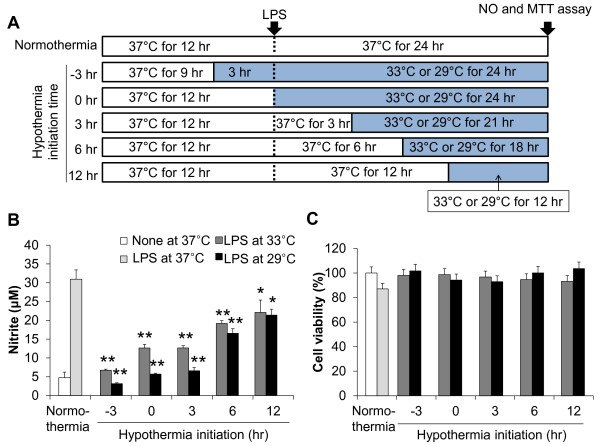
**Time-dependent effects of hypothermia on NO production in LPS-stimulated microglial cells.** Hypothermia was initiated at the different time points before or after LPS stimulation as shown in the experimental scheme (**A**). BV-2 microglial cells (5 x 10^4^ cells/well in a 96-well plate) were incubated with LPS (100 ng/ml) under hypothermic (33 °C, 29 °C) or normothermic (37 °C) conditions as indicated. The amount of nitrite in the culture media was measured with the Griess reagent at 24 hours after LPS stimulation (**B**). Cell viability was examined by MTT assays. The results represent the percentage of surviving cells compared to the control (**C**). Results are the mean ± SD (n = 3). *P < 0.05, **P < 0.01; significantly different from the LPS-treated group under normothermia (37 °C). LPS, lipopolysaccharide; MTT, 3-(4, 5-dimethylthiazol-2-yl)-2, 5-diphenyltetrazolium bromide; n, number.

**Figure 3:**
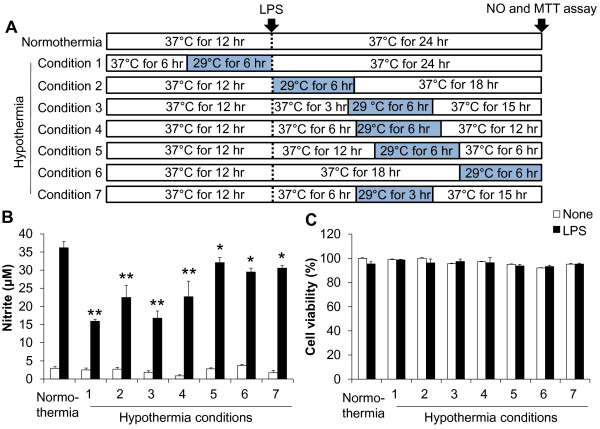
**Effects of delayed hypothermia on NO production in LPS-stimulated microglial cells.** Hypothermia was initiated at the different time points with the same duration as indicated in the experimental scheme (**A**). BV-2 microglial cells (5 x 10^4^ cells per well in a 96-well plate) were treated with LPS (100 ng/ml) for 24 hours under normothermic (37 °C) or hypothermic (29 °C) conditions as indicated (conditions 1 to 7). The concentration of nitrite in the culture media was measured with the Griess reagent at the end of the incubation (**B**). Cell viability was examined by MTT assays and the results were expressed as the percentage of surviving cells over the controls (**C**). Results are the mean ± SD (n = 3). *P < 0.05, **P < 0.01; significantly different from LPS treatment under normothermia. LPS, lipopolysaccharide; MTT, 3-(4, 5-dimethylthiazol-2-yl)-2, 5-diphenyltetrazolium bromide; n, number.

To determine the time-dependent effect of hypothermia, hypothermic exposure was initiated at different time points before or after LPS stimulation in BV-2 microglial cells (Figure 2A). BV-2 microglial cells were exposed to hypothermia (33 °C, 29 °C) three hours prior to LPS treatment or 0 to 12 hours after LPS stimulation, and NO production was assessed at the time point indicated. The results showed that all of the hypothermic treatments decreased NO production significantly, and the early-treatment showed the greatest effects (Figure 2B). The effect of 29 °C was again greater than that of 33 °C. When the initiation of hypothermia was delayed to 3 to 12 hours after LPS stimulation, the inhibitory effect was still significant. Cell viability was not significantly affected by those treatments (Figure 2C). Based on these results, further hypothermic experiments were done at 29 °C. In these experiments (Figure 2), however, both the initiation and the duration of hypothermia were different among the experimental groups. Thus, in the next set of experiments, the duration of hypothermia was fixed to six hours with different initiation schedules (Figure 3A). Hypothermia initiated six hours prior to LPS stimulation or 0 to 6 hours after LPS stimulation (conditions 1, 2, 3, and 4) efficiently inhibited microglial NO production. However, the effect of hypothermia delayed to 12 to 18 hours after LPS stimulation (conditions 5 and 6) was greatly reduced. Hypothermia for six hours duration seems to be required for the optimal inhibitory effects, since a three hour duration initiated at the same time point showed less effect (conditions 4 versus 7). There was no significant difference in cell viability under any of these conditions (Figure 3C). The results suggest that therapeutic hypothermia initiated even three to six hours after brain injury may have anti-microglia activity.

### Hypothermia inhibition of proinflammatory cytokine/chemokine expression and ROS production in microglia

**Figure 4:**
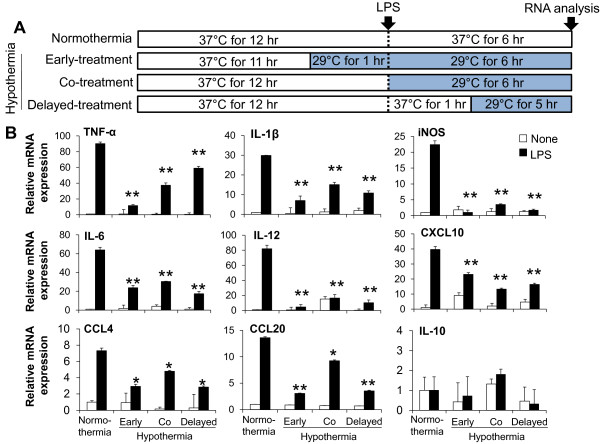
**Effect of hypothermia on pro-inflammatory gene expression in LPS-stimulated microglial cells.** Primary microglial cells (5 x 10^5^ cells per well in a six-well plate) were treated with LPS (100 ng/ml) under hypothermia (29 °C) or normothermia (37 °C) as indicated (**A**). Total RNA was isolated at six hours after the LPS treatment. The levels of cytokine or chemokine mRNA were determined by real-time RT-PCR. The data were normalized to GAPDH (**B**). Results are mean ± SD (n = 3). *P <0.05, **P < 0.01; significantly different from the LPS-treated group under normothermia. LPS, lipopolysaccharide; n, number.

**Figure 5:**
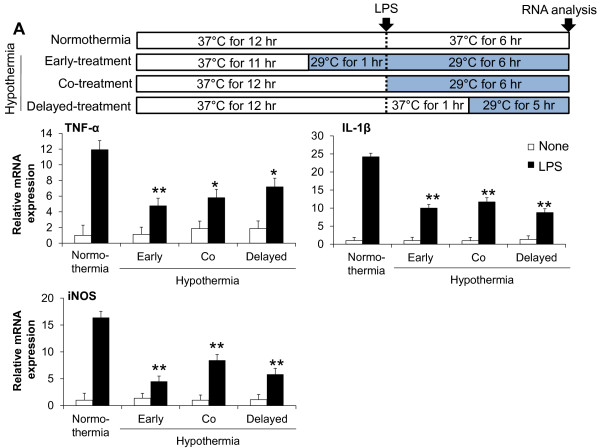
**Effect of hypothermia on pro-inflammatory gene expression in LPS-stimulated astrocyte cultures.** Primary astrocyte cultures (5 x 10^5^ cells per well in six-well plates) were treated with LPS (100 ng/ml) under hypothermia (29 °C) or normothermia (37 °C) as indicated (**A**). Total RNA was isolated at six hours after the LPS treatment. The levels of cytokine mRNA were determined by real-time RT-PCR. The data were normalized to GAPDH (**B**). Results are mean ± SD (n = 3). *P < 0.05, **P < 0.01; significantly different from the LPS-treated group under normothermia. LPS, lipopolysaccharide; n, number.

**Figure 6:**
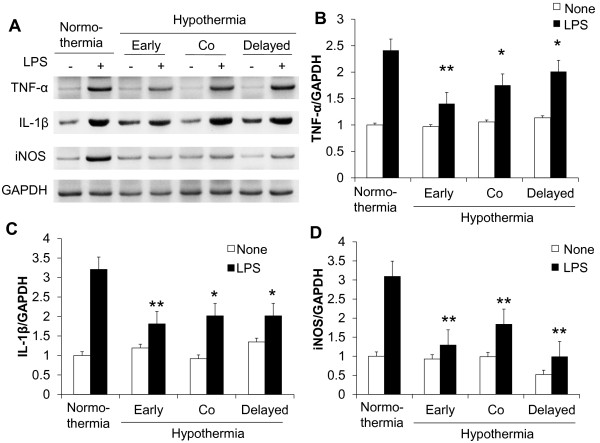
**Effect of hypothermia on pro-inflammatory gene levels in LPS-stimulated microglial cells: conventional RT-PCR analysis.** Primary microglial cells (5 x 10^5^ per well in six-well plates) were treated with LPS (100 ng/ml) under hypothermia (29 °C) or normothermia (37 °C) as indicated in the main text. Total RNA was isolated at six hours after the LPS treatment. The levels of cytokine and iNOS mRNA were determined by conventional RT-PCR (**A**). The data were normalized to GAPDH (**B**, **C**, and **D**). Results are mean ± SD (n = 3). *P < 0.05, **P <0.01; different from LPS-treated group under normothermia. LPS, lipopolysaccharide; n, number.

**Figure 7:**
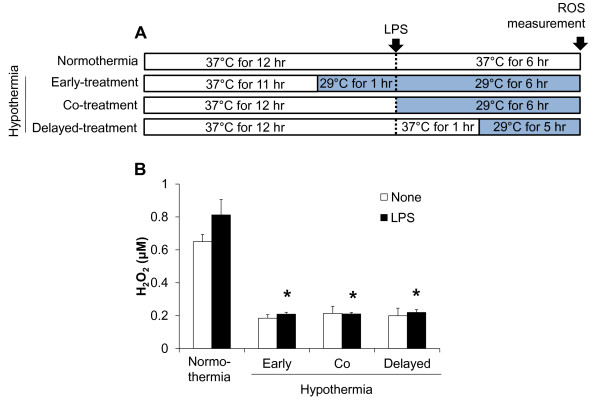
**Effect of hypothermia on ROS production in LPS-stimulated microglial cells.** BV-2 microglial cells (5 x 10^4^ cells per well in a 96-well plate) were treated with LPS (100 ng/ml) for six hours under normothermic (37 °C) or hypothermic (29 °C) conditions as indicated (**A**). Production of H_2_O_2_ was evaluated by Amplex Red assay (**B)**. Results are the mean ± SD (n = 3). *P <0.05; significantly different from the LPS-treated group under normothermia. LPS, lipopolysaccharide; n, number; ROS, reactive oxygen species.

**Figure 8:**
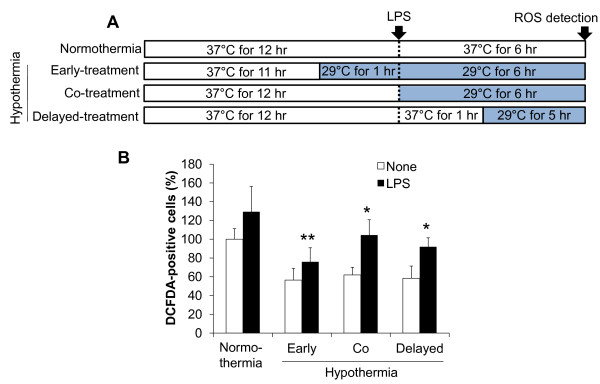
**Effect of hypothermia on ROS production in LPS-stimulated microglial cells: DCFDA assay.** BV-2 microglial cells (5 x 10^4^ cells per well in a 96-well plate) were treated with LPS (100 ng/ml) for six hours under normothermic (37 °C) or hypothermic (29 °C) conditions as indicated (**A**). Production of ROS was evaluated by using DCFDA staining (**B**). Results are mean ± SD (n = 3). *P < 0.05, **P < 0.01; significantly different from LPS treatment under normothermia. DCFDA, 2'-7'-dichlorodihydrofluorescein diacetate; LPS, lipopolysaccharide; n, number; ROS, reactive oxygen species.

**Figure 9:**
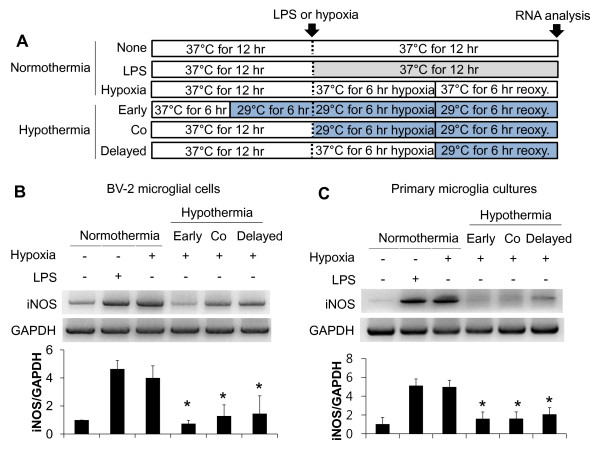
**Effect of hypothermia on iNOS gene expression in hypoxia-stimulated microglial cells.** BV-2 microglial cells or primary microglial cells (5 x 10^5^ per well in six well plates) were exposed to LPS (100 ng/ml) or hypoxia under hypothermia (29 °C) or normothermia (37 °C) as described (**A**). Total RNA was isolated at six hours after LPS or hypoxic stimulation followed by reoxygenation. The levels of iNOS mRNA were determined by conventional RT-PCR. The data was normalized to GAPDH (**B**, **C**). Results are the mean ± SD (n = 3). *P < 0.05; significantly different from the hypoxia-treated group under normothermia. iNOS, inducible NO synthase; LPS, lipopolysaccharide; n, number.

Having shown that hypothermia inhibits microglial NO production, we next determined whether hypothermia blocks microglial expression of proinflammatory cytokines, chemokines, or iNOS by Real-Time quantitative PCR (Figure 4). Early-, co-, or delayed-hypothermia attenuated with varying magnitudes the LPS-induced expression of TNF-α, IL-1β, iNOS, IL-6, IL-12, CXCL10, CCL4, and CCL20 in primary microglia cultures. It should be noted that delayed hypothermia was effective in inhibiting the expression of all proinflammatory genes examined. Anti-inflammatory cytokine IL-10 expression, however, was not significantly influenced by either LPS or hypothermia. Hypothermia inhibited astrocytic expression of proinflammatory mediators in a similar fashion (Figure 5). Hypothermia inhibition of the inflammatory gene expressions in microglial cells was similarly observed in conventional RT-PCR (Figure 6). LPS-stimulated microglial cells are known to produce reactive oxygen species (ROS). Measurements of ROS using Amplex Red (Figure 7) or DCFDA (Figure 8) revealed that hypothermia reduced microglial ROS production. Early-, co-, or delayed-hypothermia similarly reduced ROS production in microglial cells. We next employed a hypoxia model to further evaluate the effect of hypothermia on microglial activation. Hypoxia led to microglial activation as determined by iNOS induction, which was attenuated under hypothermic conditions (Figure 9). Early-treatment was most effective in inhibiting iNOS induction. These results indicate that hypothermia attenuates the inflammatory activation of microglia following LPS or hypoxic stimulation.

### The protective effect of hypothermia against microglial neurotoxicity

**Figure 10:**
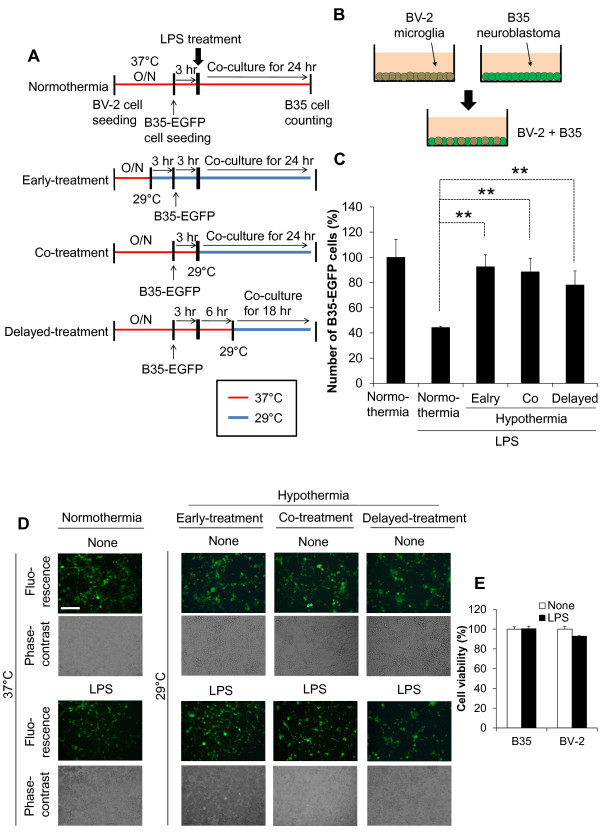
**Protective effect of hypothermia against microglial neurotoxicity.** Microglia/neuroblastoma co-culture scheme (**A, B**). BV-2 microglial cells were seeded in triplicate at a density of 1.5 x 10^4^ cells per well in a 96-well plate. B35 neuroblastoma cells (3.75 x 10^4^ cells per well) stably expressing EGFP were added onto the BV-2 microglial cells three hours prior to LPS (100 ng/ml) treatment. The co-culture of microglia and neuroblastomas was performed under normothermic or hypothermic conditions as indicated. At the end of the co-culturing, the EGFP-positive cells were counted under a fluorescence microscope to evaluate B35 neuroblastoma cell survival (**C**). **P < 0.01; different from the LPS-treated group under normothermia. LPS alone did not affect either B35 or BV-2 cell viability (**E**). Results are the mean ± SD (n = 3). Representative microscopic images are shown (**D**). Scale bar = 200 μm. EGFP, enhanced GFP; LPS, lipopolysaccharide; n, number; O/N, overnight.

**Figure 11:**
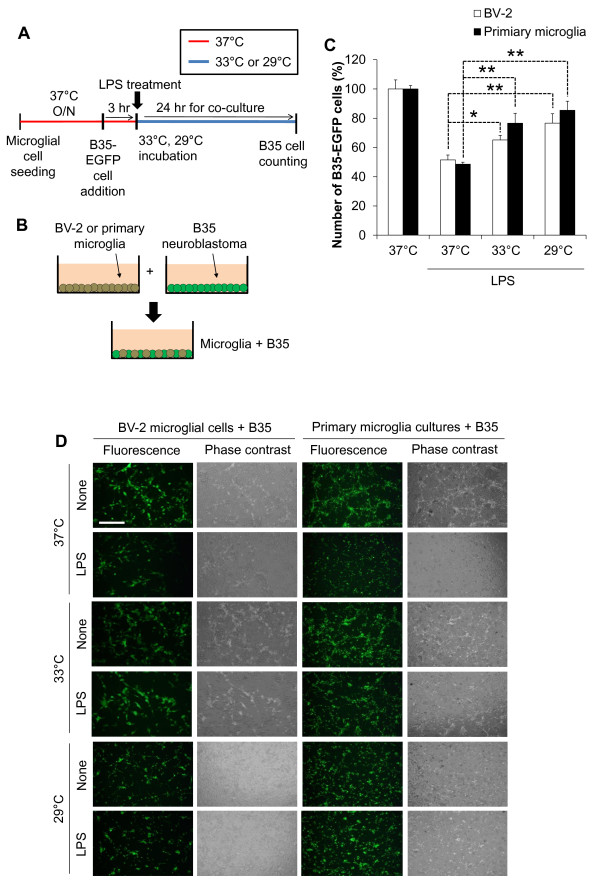
**Protective effect of hypothermia against microglial neurotoxicity: co-cultures of primary microglia and neuroblastoma cells.** B35-EGFP neuroblastoma cells were added onto BV-2 microglial cells or primary microglia cultures three hours prior to LPS (100 ng/ml) treatment. The co-culture of microglia and neuroblastoma was performed under normothermia (37 °C) or hypothermia (33 °C, 29 °C) as indicated (**A, B**). At the end of the co-culture, the EGFP-positive cells were counted under fluorescence microscope to evaluate B35 neuroblastoma cell survival (**C**). Results are mean ± SD (n = 3). *P < 0.05, **P < 0.01; different from LPS-treated group under normothermia. LPS alone did not affect B35 cell viability (data not shown). Representative microscopic images are also shown (**D**). Scale bar = 200 μm. EGFP, enhanced GFP; LPS, lipopolysaccharide; n, number; O/N, overnight

**Figure 12:**
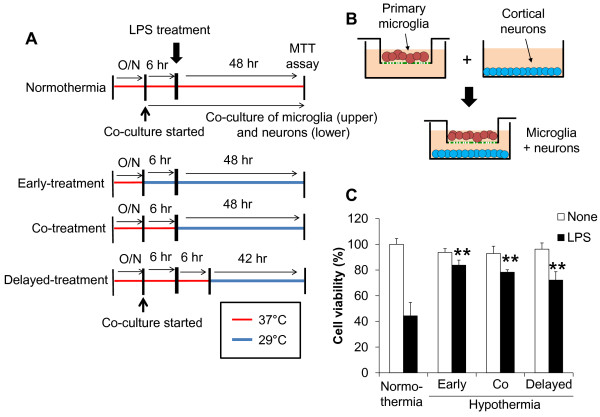
**Protective effect of hypothermia against microglial neurotoxicity in primary microglia and cortical neuron co-cultures.** Co-cultures of primary microglia and cortical neurons were done using culture inserts as shown (**A, B**). Following LPS stimulation of the primary microglia/neuron co-cultures under normothermic or hypothermic condition, the co-cultures were further incubated for 48 hours. Afterwards, the culture inserts containing microglial cells were removed, and a MTT assay was performed to determine the viability of cortical neurons (**C**). Results are the mean ± SD (n = 3). **P < 0.01; significantly different from the LPS-treated group under normothermia. LPS, lipopolysaccharide; MTT, 3-(4, 5-dimethylthiazol-2-yl)-2, 5-diphenyltetrazolium bromide; n, number; O/N, overnight.

**Figure 13:**
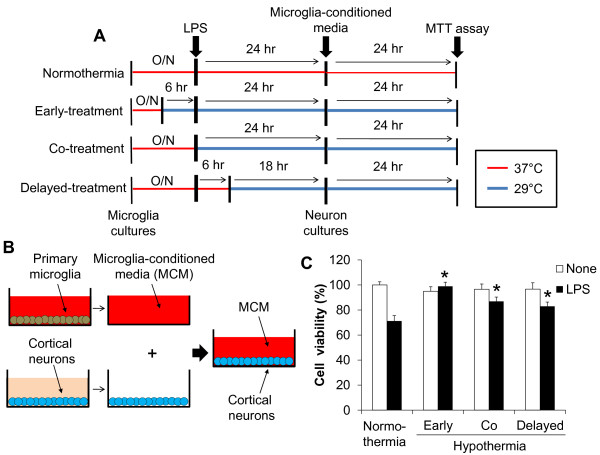
**Protective effect of hypothermia against neurotoxicity of microglia-conditioned media.** Microglia-conditioned media (MCM) were collected after stimulation of primary microglia cultures with LPS under normothermic or hypothermic condition as indicated (**A, B**). MCM were added to primary cortical neurons and the viability was assessed by a MTT assay 24 hours later (**C**). Results are mean ± SD (n = 3). *P < 0.05; different from LPS-treated group under normothermia. LPS, lipopolysaccharide; MTT, 3-(4, 5-dimethylthiazol-2-yl)-2, 5-diphenyltetrazolium bromide; n, number; O/N, overnight.

**Figure 14:**
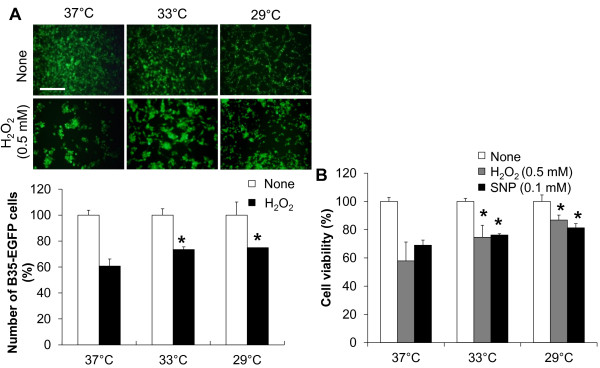
**Neuroprotective effect of hypothermia against oxidative or nitrosative stress.** B35-EGFP neuroblastoma cells were seeded in triplicate at a density of 5 x 10^4^ cells per well in 96-well plate, and then treated with H_2_O_2_ (0.5 mM) or SNP (0.1 mM) under normothermic (37 °C) or hypothermic (33 °C or 29 °C) conditions for 24 hours. After incubation B35-EGFP cells were counted (**A**, *lower*) or subjected to a MTT assay (**B**) for assessment of cell viability. Representative images of the fluorescence microscopy are shown (**A**, *upper*). Scale bar = 200 μm. Results are the mean ± SD (n = 3). *P < 0.05; significantly different from H_2_O_2_- or SNP-treated group under normothermia. EGFP, enhanced GFP; LPS, lipopolysaccharide; MTT, 3-(4, 5-dimethylthiazol-2-yl)-2, 5-diphenyltetrazolium bromide; n, number; SNP, sodium nitroprusside.

The effect of hypothermia on microglial neurotoxicity was evaluated using co-cultures of microglia and neurons. First of all, co-cultures of BV-2 microglial cells and B35-EGFP neuroblastoma cells were utilized to evaluate microglial neurotoxicity (Figure 10). In these co-cultures, LPS-stimulated microglia exhibited cytotoxic effects on the neuroblastoma cells. At the end of co-culturing, EGFP-positive viable neuroblastoma cells were counted to measure the microglial cytotoxicity toward the co-cultured neuroblastoma cells. Hypothermia given before, during, or after LPS stimulation of the microglia attenuated the microglial neurotoxicity in the co-cultures. The protective effect of hypothermia against microglial neurotoxicity was similarly observed at 33 °C and 29 °C, as well as in the co-cultures of primary microglia/neuroblastoma cells (Figure 11). The effect of hypothermia on microglial neurotoxicity was also examined in the co-cultures of primary microglia and primary cortical neurons using a culture insert (Figure 12). Primary microglia cultures were seeded in the culture insert, while primary cortical neurons were cultured in the lower compartment. Early-, co-, or delayed-treatment of hypothermia protected neurons from microglial toxicity with different magnitudes, with early treatment being most effective. Similar levels of protection were seen when conditioned media of microglia cultures were added to cortical neuron cultures (Figure 13). The direct neuroprotective effect of hypothermia was next examined by exposing B35-EGFP neuroblastoma cells to H_2_O_2_ or NO donor SNP under hypothermic conditions (Figure 14). Hypothermic conditions (33 °C or 29 °C) partly protected the neuroblastoma cells against H_2_O_2_ or SNP toxicity. These results indicate that the neuroprotective effects of hypothermia are achieved by inhibiting neurotoxic microglial activation as well as by reducing direct neurotoxicity of oxidative or nitrosative stress.

### The inhibitory effect of hypothermia on microglial cell migration

**Figure 15:**
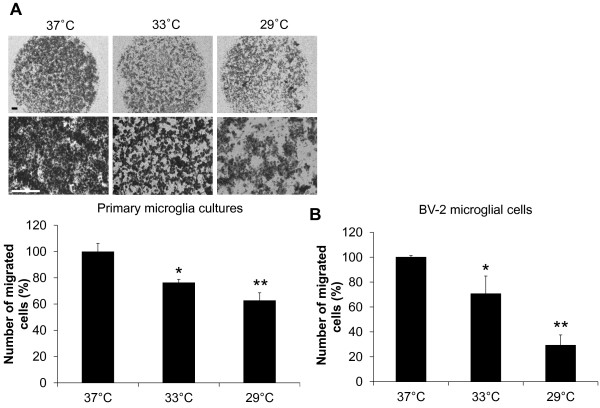
**Effect of hypothermia on microglial cell migration: the Boyden chamber assay.** Primary microglial cultures (**A**) or BV-2 microglial cells (1 x 10^4^ cells per well) (**B**) were seeded on the upper compartment of the Boyden chamber. After three hours (BV-2 microglial cells) or six hours (primary microglia) incubation under normothermic (37 °C) or hypothermic (33 C, 29 C) conditions, cells that migrated through the membrane were stained (**A** upper) and counted to evaluate the relative cell migration (**A** lower, **B**). A representative microscopic image for each condition is shown (magnification, x40 or x200) (**A** upper). Scale bar = 200 μm. Results are the mean ± SD (n = 3). **P < 0.01, *P < 0.05; significantly different from normothermia (37 C). n, number.

**Figure 16:**
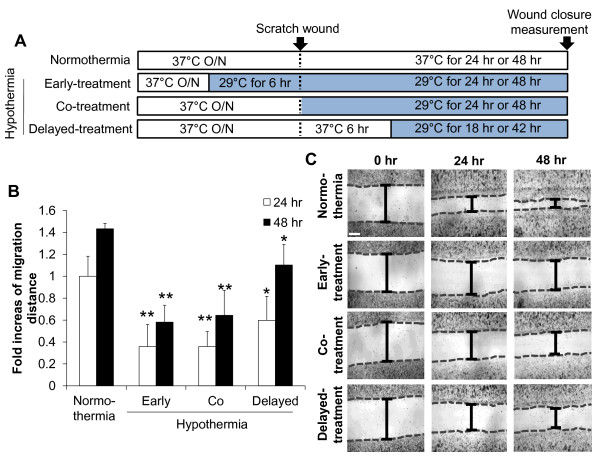
**Effect of hypothermia on microglial cell migration: wound healing assay.** Effect of hypothermia on microglial cell migration was investigated by the wound healing assay. When the cells reached 90 % confluence, a single wound was made in the center of the cell monolayer and cell debris was removed by washing with PBS. After 24 hours or 48 hours of incubation under normothermic or hypothermic condition as indicated (**A**), the wound closure areas were visualized under an inverted microscope and quantified (**B**). Results are the mean ± SD (n = 3). *P < 0.05, **P < 0.01; different from normothermia (37 °C) at the same time point. A representative microscopic image for each condition is shown (magnification, x100) (**C**). Scale bar = 200 μm. n, number; O/N, overnight.

**Figure 17:**
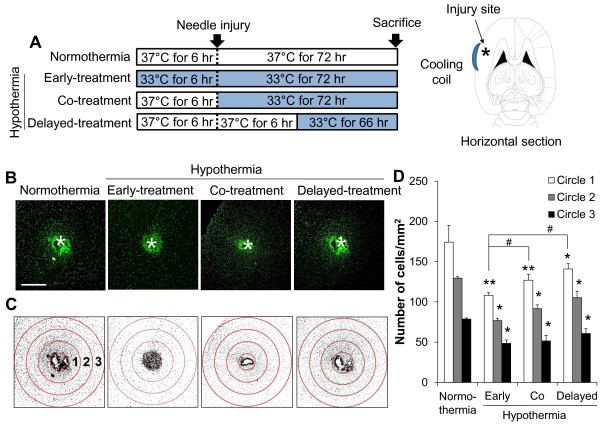
**Effect of hypothermia on microglial migration*in vivo*.** To assess the effect of therapeutic hypothermia on microglial migration *in vivo*, focal stab injury was created by placing a needle in the cortical area of the brain through a guide cannula. Local hypothermia (33 °C) using cold water circulation through a cooling coil was initiated either six hours before or after the needle injury, and maintained for 66 to 78 hours as indicated (**A**). Asterisks indicate the injury site. Horizontal sections of rat brain were subjected to microglial immunohistochemistry (Iba-1, green) (**B**). A representative microscopic image for each condition is shown (magnification, x40). Scale bar = 1 mm. Data acquisition and immunohistological intensity measurement of Iba-1 staining was performed with a NIH image J program (**C**). To count the Iba-1 positive cells, three concentric circles with constant interval were placed in the peri-region of the injury site (asterisk) in the sub-threshold image of the six independent sections. Three animals were used for each experimental group. The cells in the three circles were counted and statistically analyzed (**D**). Results are the mean ± SD. *P < 0.05, **P < 0.01; different from the same circle under normothermia (37 °C). #*P* < 0.05; different from the two conditions indicated.

**Figure 18:**
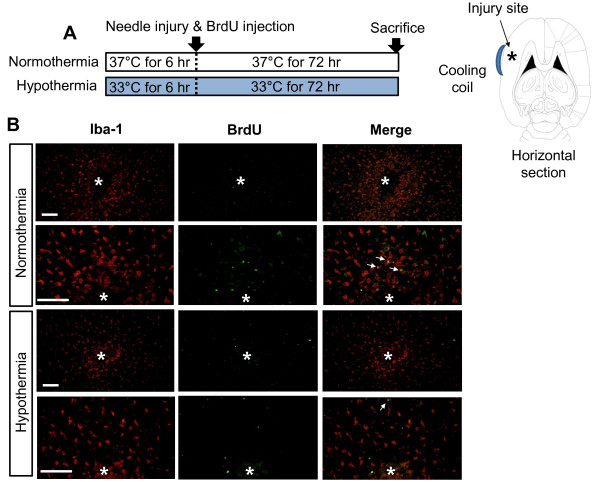
**Effects of local hypothermia on microglial proliferation*in vivo*.** A focal needle injury and BrdU injection (i.p., 200 mg/kg) were done under normothermic or hypothermic condition as indicated (**A**). Iba-1 (red) or BrdU (green) immunochemistry was done to determine the effects of local hypothermia on microglial proliferation *in vivo* (**B**). Asterisks indicate the injury site. Arrows indicate Iba-1 and BrdU double positive cells in the merged images. A representative microscopic image for each condition is shown (magnification, x200 or x400). Scale bar = 200 μm. BrdU, 5-bromo-2’-deoxyuridine; i.p., intraperitoneally.

**Figure 19:**
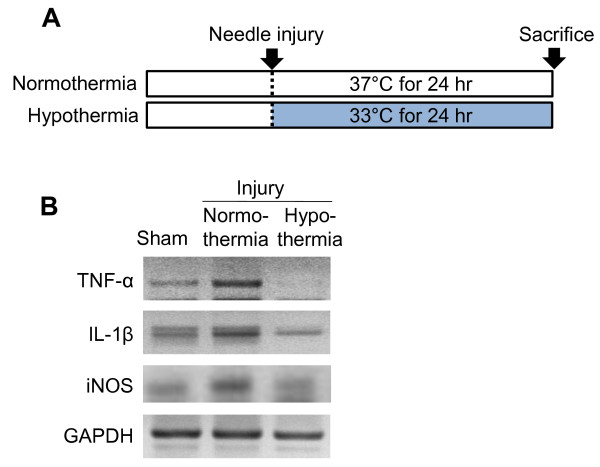
**Inhibition of proinflammatory cytokine and iNOS expression by local hypothermia in the rat model of stab injury.** After the focal stab injury in the cortex under normothermia or hypothermia as described in the main text (**A**), cortical tissues were prepared and subjected to RT-PCR analysis (**B**). Compared to normothermia, hypothermia diminished the injury-induced expression of TNF-α, IL-1β, and iNOS. iNOS, inducible NO synthase.

It has been previously shown that microglial cell migration is one of the main components of brain responses to injury and inflammation [45]. The effect of hypothermia on microglial cell migration was determined by two different cell migration assays. In the Boyden chamber assay, the migration of BV-2 microglial cells or primary microglial cells across a membrane was diminished by hypothermic conditions (33 °C or 29 °C) compared to normothermia (Figure 15). The migration-inhibiting effects of hypothermia were greater at 29 °C than at 33 °C. Similar effects of hypothermia (29 °C) were observed in the wound healing assay (Figure 16). After the BV-2 microglial cells were exposed to hypothermia before or after the scratch wound, microglial cell migration was assessed by measuring wound closure at the end of a 24 to 48 hour incubation period. Early-, co-, and delayed-treatment of hypothermia suppressed the wound closure to different degrees. No significant difference in cell proliferation was observed between normothermia and hypothermia conditions during this time period (data not shown). Delayed hypothermia effectively inhibited microglial migration, albeit to a lesser degree than that of early- or co-treatment. These results indicate that delayed hypothermia may reduce microglial migration in inflammatory brain. In the next set of experiments, the effect of hypothermia on microglial migration was examined *in vivo* using a rat model of stab injury (Figure 17). Local hypothermia (33 °C) was applied to rat brain using a cooling coil before, during, or after a needle injury in the cortex, and then migration of microglia toward the injury site was evaluated by immunohistochemical staining of Iba-1, a microglia-specific marker. Immunohistochemical image analysis of horizontal sections revealed that local hypothermia significantly inhibited microglial migration toward the injury site. Relative migration of microglia was based on the number of cells present in the three concentric circles (Figure 17C). Proliferation of microglia was minimal around the injury site as determined by BrdU staining (Figure 18), indicating that the increase in the number of microglia around the injury site was mostly due to migration rather than proliferation of microglia. A similar result was obtained from PCNA staining (data not shown). RNA analysis of cortical tissue indicated that local hypothermia also attenuated the injury-induced expression of proinflammatory cytokines such as TNF-α and IL-1β and iNOS (Figure 19), corroborating the earlier findings on the anti-inflammatory effects of hypothermia *in vivo*.

## Discussion

In this study, we report that hypothermia reduces microglial activation in a cooling time-dependent manner. Hypothermia attenuated several phenotypes of microglia that are associated with neuroinflammation: 1) hypothermia inhibited microglial production of NO, ROS, and proinflammatory cytokines/chemokines following TLR4 or hypoxic stimulation; 2) it attenuated microglial neurotoxicity; and 3) it reduced microglial migration toward the injury site. More importantly, hypothermia initiated after stimulation of the microglia (with either LPS or hypoxia) was also able to inhibit inflammatory activation of microglia, and the inhibitory effects of hypothermia were initiation and duration time-dependent. This was demonstrated in the microglial production of inflammatory mediators, microglial neurotoxicity, and microglial migration. In general, the microglia-inhibiting effects of hypothermia were greater at 29 °C than at 33 °C, although the differences between the two temperatures seemed small in some cases.

Our data suggest that post-injury hypothermia may effectively suppress deleterious microglial activation and subsequent neuroinflammation. Our observation that hypothermic exposure given after microglial stimulation is effective in attenuating inflammatory activation of microglia has important clinical implications, since the therapeutic hypothermia is usually delivered to patients after brain damages, such as ischemic stroke and traumatic brain injury, to improve neurological outcomes as one of the neuroprotective strategies [46]. We have also demonstrated using cultured microglial cells as an *in vitro* model that hypothermia initiated three hours after inflammatory stimulation (six hours duration) most strongly inhibited microglial NO production among the different co- or delayed hypothermic conditions: in fact, it was as effective as hypothermia initiated before inflammatory stimulation (pre-LPS hypothermia) (Figure 3). These results may help determine the optimal time window for post-injury hypothermic therapy. It was previously reported that hypothermic preconditioning prior to injury was effective through tolerance induction in a mouse model [47,48]. In the present study, pretreatment by hypothermia also showed superior effects in general including reduction in microglial NO/TNF-α production, neurotoxicity, and cell migration. These results may be attributed to the preconditioning effects or the longer duration of the hypothermia treatment [27,49-51]. Our data indicate that hypothermia initiated six hours after LPS stimulation efficiently inhibited microglial nitrite production. In the report by Ohta *et al*., the therapeutic time window of hypothermia lasted for four hours after reperfusion in a middle cerebral artery occlusion (MCAO) model [52]. Hypothermia was effective when initiated 30 minutes to three hours after occlusion/reperfusion in the MCAO model in other studies [53-62]. This indicates that the maximum therapeutic time window of hypothermia may be four to six hours after injury. Our results showed that LPS-induced pro-inflammatory cytokine mRNA levels were downregulated by hypothermia in microglial cells, which is consistent with previous studies [15,18,20,38,63]. Lowering the temperature decreased p38 MAPK activation and the subsequent p38-regulated production of proinflammatory cytokines and NO in ATP-activated microglia [17], suggesting that the suppression of p38 is one of the possible mechanisms of hypothermic attenuation of proinflammatory gene expression in microglia. Although delayed hypothermia suppressed cytokine mRNA expression more effectively than co-treatment, TNF-α showed a different mRNA expression pattern from the others (Figure 4). The differential effects of hypothermia on the expression of TNF-α and other proinflammatory mediators were also observed in the conventional RT-PCR analysis (Figure 6) and in previous reports that used a variety of experimental models [18,64,65]. It is well documented that the gene expression of TNF-α is regulated at the post-transcriptional level, such as mRNA stability [66-68]. Measurement of TNF-α and other cytokines at the protein level should be done in future studies to further understand the differential effects of hypothermia. There was no difference between early-, co-, and delayed-hypothermia on the production of H_2_O_2_ (Figure 7). While the Amplex Red assay primarily detects H_2_O_2_ released from the cells, the cell permeant fluorogenic dye, DCFDA, measures hydroxyl, peroxyl, and other ROS activity within the cell. Our data indicate that hypothermia effectively blocked H_2_O_2_ released from microglia, no matter when the hypothermia was initiated (as determined by Amplex Red in Figure 7). However, early-, co-, and delayed hypothermia showed differential effects on intracellular ROS production (as determined by DCFDA in Figure 8). As the inhibitory potency of hypothermia on neural cell death was similar among the different cellular assay systems (Figures 10,11,12, and 13), the neurotoxicity may be mainly caused by soluble toxic factors. Previous studies showed that TNF-α, nitric oxide, IL-1β, IL-6, MCP-1 glutamate, prostaglandins, and ROS are responsible for neurotoxicity [38,69-71].

In the brain injury region, activated microglia cells are presumed to migrate toward the tissue damage and transform into phagocytes [72,73]. Effects of hypothermia on the migration and phagocytic activity of the phagocytes have been previously investigated. Migration of human polymorphonuclear cells towards a chemotactic stimulus and their capacity for superoxide anion production was significantly reduced at 30 °C. In another study, the phagocytic capacity of neutrophils for *Staphylococcus aureus* was impaired at 29 °C [74], and pig neutrophils and monocyte migration *in vivo* were also reduced at 29 °C [75]. Moreover, a previous study showed that moderate hypothermia resulted in a reduction in IL-1β-induced leukocyte rolling and adhesion in the pial microvasculature of mice, providing neuroprotection [76]. Hypothermia downregulated the expression of migration inhibitory factor (MIF) in a rat model of traumatic brain injury [77]. Also, in an ischemia model, both intraischemic and delayed hypothermia decreased intercellular adhesion molecule-1 (ICAM-1) expression [78], which might play a significant role in cell extravasation and migration [79]. These previous findings are in agreement with the current results that hypothermia attenuates microglial migration in the Boyden chamber assay, wound healing assay, and rat stab injury model.

## Conclusions

Our results show that therapeutic hypothermia exerts time-dependent effects on microglial activation and migration. Delayed hypothermia initiated after the inflammatory stimulation of the microglia in culture was able to attenuate the production of inflammatory mediators, thereby indicating that post-injury or -ischemic hypothermia may be therapeutically relevant to reduce deleterious neuroinflammation and secondary brain injuries.

## Abbreviations

BBB, Blood–brain barrier; BrdU, 5-Bromo-2'-deoxyuridine; BSA, Bovine serum albumin; CMH2DCFDA, 5-(and 6-) chloromethyl-2’,7’-dichlorodihydrofluorescein diacetate; Cy3, Cyanine Dye 3; CXCL10, C-X-C motif chemokine 10; CCL4, Chemokine (C-C motif) ligand 4; CCL20, Chemokine (C-C motif) ligand 20; CNS, Central nerve system; DAPI, 4',6-diamidino-2-phenylindole; DMEM, Dulbecco’s modified Eagle’s medium; DMSO, Dimethyl sulfoxide; EDTA, Ethylenediamine tetraacetic acid; EGFP, Enhanced green fluorescent protein; FBS, Fetal bovine serum; FITC, Fluorescein isothiocyanate; GAPDH, Glyceraldehyde 3-phosphate dehydrogenase; HEPES, N-2-hydroxyethylpiperazine-N'-2-ethanesulfonic acid; HRP, Horseradish peroxidase; iNOS, Inducible NO synthase; IL-1β, Interleukin-1 beta; Iba-1, Ionized calcium binding adaptor molecule 1; IL-6, Interleukin-6; IL-12, Interleukin-12; LPS, Lipopolysaccharide; MCM, Microglia-conditioned media; MTT, 3-(4 5-dimethylthiazol-2-yl)-2, 5-diphenyltetrazolium bromide; NO, Nitric oxide; OCT, Optimal cutting temperature; PBS, Phosphate-buffered saline solution; PCNA, Proliferating Cell Nuclear Antigen; PFA, Paraformaldehyde; ROS, reactive oxygen species; RT-PCR, reverse transcriptase-polymerase chain reaction; SNP, Sodium nitroprusside; TNF-α, Tumor necrosis factor alpha; TLR4, Toll-like receptor 4.

## Competing interests

The authors declare that they have no competing interests.

## Authors’ contributions

J-WS performed the experiments and analyzed the data as well as wrote the manuscript. Jong-Heon Kim and Jae-Hong Kim participated in animal studies. MS, HSH, and JP participated in the study design and data interpretation. KS is the main investigator of this work in charge of the study design, analysis and interpretation of results, and writing. All authors read and approved the final manuscript.
